# Supplementary material for: MetaRibo-Seq measures translation in microbiomes
Source: Nat Commun. 2020 Jun 29;11:3268. doi: 10.1038/s41467-020-17081-z (PMC7324362; doi:10.1038/s41467-020-17081-z)
Supplement: Supplementary file 10 — Supplementary Data 7 [file 41467_2020_17081_MOESM10_ESM.zip › File2/Confidence_High_Taxonomy/57881_out.krona.html]

Javascript must be enabled to view this page.

members
magnitude
magnitudeUnassigned
count
unassigned
taxon
rank

57881\_out

186

superkingdom
186
2

976
186
phylum

class
200643
186

38
order

SRS013098\_contig\_number\_27918SRS013476\_contig\_number\_26412SRS013800\_contig\_number\_3946SRS014415\_contig\_number\_337SRS014459\_contig\_number\_34786SRS015369\_contig\_number\_3416SRS015431\_contig\_number\_14143SRS017375\_contig\_number\_contig-100\_906.18911SRS019068\_contig\_number\_29619SRS019381\_contig\_number\_10269SRS019910\_contig\_number\_24597SRS019968\_contig\_number\_15715SRS022524\_contig\_number\_7234SRS022609\_contig\_number\_25360SRS043701\_contig\_number\_contig-100\_1651.101852SRS047044\_contig\_number\_18149SRS049712\_contig\_number\_17280SRS049995\_contig\_number\_22005SRS050974\_contig\_number\_864SRS055982\_contig\_number\_contig-100\_745.120116SRS057480\_contig\_number\_10064SRS063040\_contig\_number\_24437SRS063370\_contig\_number\_11518SRS064557\_contig\_number\_13629SRS065397\_contig\_number\_2419SRS104036\_contig\_number\_7838SRS105153\_contig\_number\_3578SRS1055034\_contig\_number\_9781SRS143372\_contig\_number\_8117SRS144714\_contig\_number\_28748SRS147088\_contig\_number\_2343SRS148424\_contig\_number\_26950SRS148784\_contig\_number\_7029SRS149784\_contig\_number\_24776SRS892882\_contig\_number\_14996SRS893327\_contig\_number\_10738SRS893378\_contig\_number\_22475SRS971276\_contig\_number\_25107
171549
186

family
171552
1

838
1
genus

species

SRS075878\_contig\_number\_18483
1
165179

815
44
family

5
816
44

SRS013687\_contig\_number\_34616SRS015694\_contig\_number\_19931SRS056259\_contig\_number\_23620SRS142923\_contig\_number\_15194SRS893300\_contig\_number\_2631
genus

204516
1

SRS893172\_contig\_number\_9674
species


SRS077392\_contig\_number\_26324
species
821
1

246787
3
species

SRS045004\_contig\_number\_14151SRS101433\_contig\_number\_524SRS893252\_contig\_number\_12069

1
310297

SRS098073\_contig\_number\_2993
species

3
162156

SRS011302\_contig\_number\_21421SRS143466\_contig\_number\_32465SRS893279\_contig\_number\_15386
species


SRS098644\_contig\_number\_32735
species
1
371601


SRS046068\_contig\_number\_4935SRS148091\_contig\_number\_13739SRS893341\_contig\_number\_1470
species
2292930
3

species

SRS014683\_contig\_number\_356SRS016495\_contig\_number\_13655
2
820

species

SRS062654\_contig\_number\_17209SRS105082\_contig\_number\_19754
46506
2

5
2292953

SRS1041157\_contig\_number\_6115SRS143148\_contig\_number\_5542SRS893292\_contig\_number\_6605SRS893295\_contig\_number\_6619SRS893373\_contig\_number\_16297
species

817
3
species

SRS056537\_contig\_number\_24393SRS146813\_contig\_number\_16339SRS893270\_contig\_number\_22733

1
329854

SRS101376\_contig\_number\_20654
species


SRS019178\_contig\_number\_4361
species
1
1897051

28116
4
species

SRS013382\_contig\_number\_contig-100\_24.27381SRS142503\_contig\_number\_29497SRS893230\_contig\_number\_7744SRS893342\_contig\_number\_5766

species

SRS053214\_contig\_number\_14929SRS893366\_contig\_number\_18400
2
818

species

SRS018575\_contig\_number\_5188
1
585543

3
47678
species

SRS019397\_contig\_number\_9683SRS042966\_contig\_number\_20499SRS058723\_contig\_number\_2913

species

SRS016753\_contig\_number\_2445SRS020233\_contig\_number\_50280
2
28111

1853231
71
family

genus
4
283168

species

SRS012969\_contig\_number\_542SRS024435\_contig\_number\_5436SRS077127\_contig\_number\_3205SRS077849\_contig\_number\_144
4
28118

67
574697
genus

63
2093856

SRS014235\_contig\_number\_15457SRS014412\_contig\_number\_2976SRS014948\_contig\_number\_8212SRS015190\_contig\_number\_2566SRS016203\_contig\_number\_12462SRS016335\_contig\_number\_19196SRS016629\_contig\_number\_2656SRS016954\_contig\_number\_27176SRS018541\_contig\_number\_8875SRS018836\_contig\_number\_26596SRS018888\_contig\_number\_2028SRS019030\_contig\_number\_18751SRS019161\_contig\_number\_28118SRS023526\_contig\_number\_683SRS023914\_contig\_number\_22148SRS024492\_contig\_number\_18221SRS024663\_contig\_number\_12818SRS043001\_contig\_number\_10576SRS043411\_contig\_number\_15997SRS045739\_contig\_number\_7473SRS045826\_contig\_number\_19829SRS046717\_contig\_number\_90SRS047433\_contig\_number\_8905SRS049446\_contig\_number\_4324SRS050801\_contig\_number\_2155SRS050941\_contig\_number\_83SRS050998\_contig\_number\_13281SRS052697\_contig\_number\_29025SRS053398\_contig\_number\_2673SRS053573\_contig\_number\_1662SRS063190\_contig\_number\_10293SRS065176\_contig\_number\_14988SRS074670\_contig\_number\_6583SRS075773\_contig\_number\_14885SRS076756\_contig\_number\_21867SRS076804\_contig\_number\_12451SRS076976\_contig\_number\_35SRS077024\_contig\_number\_14119SRS077231\_contig\_number\_12106SRS077730\_contig\_number\_5275SRS098571\_contig\_number\_18693SRS1041039\_contig\_number\_5951SRS1041095\_contig\_number\_328SRS1041138\_contig\_number\_2901SRS1041143\_contig\_number\_2802SRS1041147\_contig\_number\_166SRS104400\_contig\_number\_34134SRS104693\_contig\_number\_10552SRS1054716\_contig\_number\_10927SRS1055076\_contig\_number\_11652SRS143085\_contig\_number\_2578SRS143342\_contig\_number\_9221SRS143523\_contig\_number\_16120SRS143895\_contig\_number\_28386SRS144537\_contig\_number\_40404SRS144603\_contig\_number\_547SRS147557\_contig\_number\_24394SRS148159\_contig\_number\_21280SRS148196\_contig\_number\_41957SRS148970\_contig\_number\_3739SRS149181\_contig\_number\_12187SRS149325\_contig\_number\_2963SRS893187\_contig\_number\_19252
species

species

SRS015264\_contig\_number\_18123SRS018313\_contig\_number\_11422SRS020394\_contig\_number\_11163SRS1041145\_contig\_number\_22928
4
2042309

171550
24
family

3
24
239759

SRS016381\_contig\_number\_contig-100\_43.29648SRS148817\_contig\_number\_1605SRS150029\_contig\_number\_19051
genus

1
1262693
species

SRS017916\_contig\_number\_16995

28117
5

SRS015065\_contig\_number\_6990SRS018623\_contig\_number\_3810SRS078419\_contig\_number\_3024SRS1055069\_contig\_number\_1942SRS893231\_contig\_number\_912
species


SRS1041118\_contig\_number\_15961
species
1
1896969


SRS075341\_contig\_number\_11207SRS144362\_contig\_number\_21186
species
2
2292910


SRS020622\_contig\_number\_11SRS077294\_contig\_number\_14763SRS1041038\_contig\_number\_16256SRS144135\_contig\_number\_8448SRS148319\_contig\_number\_5542
species
5
328814

214856
2
species

SRS046369\_contig\_number\_9779SRS1041033\_contig\_number\_27759

species

SRS054352\_contig\_number\_2670SRS098717\_contig\_number\_8742SRS147425\_contig\_number\_7119SRS149075\_contig\_number\_16266
4
1118061


SRS074964\_contig\_number\_5500
species
1
1262694

family
2005525
8

375288
8
genus

species

SRS056273\_contig\_number\_940
823
1

46503
6
species

SRS047014\_contig\_number\_28121SRS048164\_contig\_number\_29141SRS050752\_contig\_number\_24283SRS078242\_contig\_number\_25643SRS097920\_contig\_number\_13208SRS140513\_contig\_number\_3038

1
2293123
species

SRS147614\_contig\_number\_2007
